# Impact of Time-to-Treatment Initiation and First Inter-Cycle Delay in Patients with Hodgkin Lymphoma

**DOI:** 10.3390/jcm14124085

**Published:** 2025-06-10

**Authors:** Deniz Donmez, Yasemin Evlendi, Taha Koray Sahin, Ibrahim Barista, Serkan Akin

**Affiliations:** 1 Department of Internal Medicine, Faculty of Medicine, Hacettepe University, Sihhiye, Ankara 06230, Turkey; denizdd1994@gmail.com (D.D.); ibarista@yahoo.com (I.B.); 2 Division of Medical Oncology, Cancer Institute, Hacettepe University, Sihhiye, Ankara 06230, Turkey; dryasemen13@gmail.com (Y.E.); takorsah@gmail.com (T.K.S.)

**Keywords:** Hodgkin lymphoma, ABVD, treatment delay, beta-2 microglobulin, inter-cycle delay, DTI

## Abstract

**Background**: Delays in treatments are frequent in real-world lymphoma management. This study evaluates the impact of diagnosis-to-treatment intervals (DTIs) and first inter-cycle delay (IcD) on outcomes in patients with Hodgkin lymphoma (HL) receiving ABVD chemotherapy. **Methods**: We retrospectively analyzed 137 patients with classical HL treated with ABVD at a single institution between 2015 and 2022. **Results**: The median age was 34 years (range: 18–73), and 62% were male. The median DTI was 14 days, with 24.1% of patients experiencing a delay of >7 days between the first and second chemotherapy cycles. The most frequent reason for delay was neutropenia, observed in 69% of delayed cases. Neither DTI nor IcD was significantly associated with PFS or OS. Multivariate analysis identified elevated beta-2 microglobulin as an independent predictor of both inferior PFS and OS. **Conclusions**: This is the first study to evaluate both DTI and first IcD as independent prognostic factors in HL. Modest delays in treatment initiation or early cycle administration did not negatively affect survival. Timely but flexible scheduling of ABVD may be appropriate in HL. Prospective studies are warranted in the era of novel therapeutic agents.

## 1. Introduction

Hodgkin lymphoma (HL) accounts for approximately 10–15% of all lymphomas and remains one of the most curable malignancies with multi-agent chemotherapy [1,2]. Since the mid-1970s, the combination of doxorubicin, bleomycin, vinblastine, and dacarbazine (ABVD) has been the cornerstone of treatment for all disease stages [3]. In recent years, novel agents such as brentuximab vedotin and immune checkpoint inhibitors have been incorporated into frontline treatment, especially for patients with advanced stages [4,5].

The diagnosis-to-treatment interval (DTI) is the period between histopathologic confirmation and initiation of therapy. Although longer DTIs are sometimes necessitated by additional staging or diagnostic procedures, studies suggest that shorter DTIs are paradoxically associated with worse outcomes in aggressive lymphomas, reflecting higher tumor burden and poor prognostic features [6,7]. For instance, short DTI has been correlated with elevated circulating tumor DNA and adverse clinical features, even after adjustment for conventional risk scores [7]. Moreover, a recent study from Japan suggested that short DTI (0–22 days) had a poorer outcome than long DTI (over 22 days) [8]. In contrast, some studies reported reverse results on the influence of DTI in patients with DLBCL [9,10]. However, these findings primarily derive from studies of diffuse large B-cell lymphoma (DLBCL), and the clinical relevance of DTI in HL remains underexplored.

In addition to delays in initiating treatment, interruptions or delays between chemotherapy cycles, referred to as inter-cycle delays (IcDs), are commonly encountered [11]. These delays may result from treatment-related toxicities or logistical challenges in healthcare delivery. IcDs, especially early in the treatment course, have been associated with inferior outcomes in solid tumors [12]. Nevertheless, their relevance in HL remains unclear. This study aims to assess the association of DTI and first IcD on clinical outcomes in patients with HL receiving ABVD chemotherapy.

## 2. Materials and Methods

### 2.1. Study Population

This was a retrospective single-center study conducted on patients with HL who were >18 years old and received ABVD between January 2015 and December 2022. Eligible patients were required to have histologically confirmed Hodgkin lymphoma, be aged 18 years or older, and have received at least one cycle of ABVD chemotherapy. Patients with prior malignancies, primary refractory disease, or incomplete treatment records were excluded from the analysis. Demographic and clinical data were retrieved from institutional electronic medical records. Variables collected included age, sex, disease stage, presence of B symptoms, Eastern Cooperative Oncology Group (ECOG) performance status, and baseline laboratory parameters such as lactate dehydrogenase levels. Information on treatment initiation date, chemotherapy administration dates, and disease status at last follow-up was also recorded. The study was approved by the Hacettepe University Faculty of Medicine’s Research Ethics Committee (GO22/1142).

### 2.2. Statistical Analyses

The outcome was evaluated using progression-free survival (PFS) and overall survival (OS). PFS was calculated as the time from diagnosis to the date of progression, relapse, last follow-up, or death. OS was calculated as the time from diagnosis to the date of death or last follow-up. Patients not experiencing an event were censored at their last known follow-up. DTI was calculated as the interval time in days between the reported date of final diagnosis and the initiation of ABVD. The association between DTI and the clinical outcomes of patients was assessed. The first IcD was computed as the number of extra days beyond the normal interval of two weeks between the first and second cycles of ABVD. The link between the first IcD and the outcomes was also assessed. The Kaplan–Meier method was used to depict the PFS and OS curves, and the log-rank test was implemented to evaluate the survival differences between groups. Following univariate regression analysis, variables with *p* < 0.20 and other variables considered clinically significant were selected for multivariate regression analysis. Cox regression analyses were carried out for the univariate and multivariate analyses for PFS and OS, and hazard ratios (HRs) and their 95% confidence intervals (CIs) were calculated. Statistical analyses were performed using SPSS version 25.0 (IBM Inc., Armonk, NY, USA), with statistical significance defined as a *p*-value of less than 0.05.

## 3. Results

### 3.1. Patient Characteristics

A total of 137 patients were identified, with a median age of 34 years (range 18–73). Eighty-five patients (62%) were male, and most patients (93%) had an ECOG-PS of 0 or 1. There were more patients with early-stage disease than patients with advanced-stage disease (53% vs. 47%). Among the patients with early disease stages, 54 were favorable. Table 1 presents the baseline characteristics of the patients.

### 3.2. DTI and IcD Results

The median DTI was 14 days (IQR: 7–27; min–max: 1–47). When we divided patients into two groups based on DTI, the short DTI (0–14 day) cohort included 69 patients (51%) with a median DTI of 7 days (min–max: 1–14), while the long DTI (>14 day) cohort included 68 patients (49%) with a median DTI of 27 days (min–max: 15–47). The median IcD was 0 day (IQR: 0–6; min–max: 0–28). Of the 137 patients, 72 (52.6%) did not experience any treatment delay in the second cycle of ABVD. Although the cause of treatment delay was not fully recorded, neutropenia was identified in 45 of 65 patients; this was the most common cause of delay. Neutropenic fever was detected in only three of these patients. Other complications included bacterial pneumonia in two patients, COVID-19 infection in six patients, and thrombocytopenia in one patient. Treatment was delayed for unknown reasons in 11 patients, and 33 patients (24.1%) experienced treatment delay of >7 days.

### 3.3. Survival Outcomes

**Figure 1:**
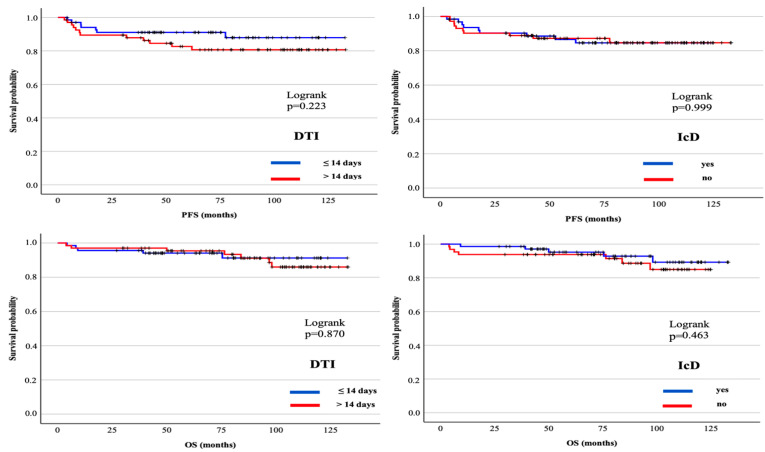
Progression-free survival and overall survival for DTI and IcD.

At a median follow-up of 81.9 months (95% CI: 75.1–85.9 months), the median PFS and OS were not reached for all patients. There were no significant differences in PFS and OS between DTI cohorts. Likewise, the presence of the first IcD did not have any significance in PFS and OS (Figure 1). There were 12 deaths (8.8%) in the entire study cohort. All deaths were due to disease progression.

In univariate analysis, younger age (HR: 0.964, 95% CI: 0.929–1.0; *p* = 0.05), elevated erythrocyte sedimentation rate (ESR) (HR: 1.013, 95% CI: 1.001–1.024; *p* = 0.03), and decreased Hb (HR: 0.781, 95% CI: 0.631– 0.968; *p* = 0.024) were associated with worse PFS. In univariate analysis for OS, older age (HR: 1.095, 95% CI: 1.048–1.143; *p* < 0.001), decreased levels of hemoglobin, lymphocyte count, and albumin ((HR: 0.697, 95% CI: 0.523–0.929; *p* = 0.014), (HR: 0.378, 95% CI: 0.145–0.982; *p* = 0.046), and (HR:0.202, 95% CI: 0.098–0.417; *p* < 0.001), respectively), and elevated levels of beta-2 microglobulin (HR: 1.001, 95% CI: 1.001–1.001; *p* < 0.001) were associated with inferior survival. We found no statistically significant impact of DTI and first IcD in both PFS ((HR: 1.022, 95% CI: 0.987–1.058; *p* = 0.218) and (HR: 0.960, 95% CI: 0.858–1.073; *p* = 0.471), respectively), and OS ((HR: 0.996, 95% CI:0.950–1.045; *p* = 0.878) and (HR: 1.028, 95% CI: 0.928–1.138; *p* = 0.596), respectively).

In multivariable analysis, a higher level of Beta-2 microglobulin was associated with worse PFS and OS (HR: 1.001, 95% CI: 1.000–1.002; *p* = 0.018 for PFS and HR: 1.001, 95% CI: 1.000–1.001; *p* = 0.037 for OS). Although younger age at diagnosis was associated with worse PFS, it had superior OS than older age (HR: 0.951, 95% CI: 0.908–0.996; *p* = 0.034 for PFS and HR: 1.061, 95% CI: 1.000–1.125; *p* = 0.049 for OS). Table 2 and Table 3 represent the factors that were associated with PFS and OS in the multivariable analysis, respectively.

## 4. Discussion

Our results revealed that neither a shorter DTI nor a prolonged delay between the first and second cycles of treatment was associated with adverse outcomes in terms of PFS or OS in HL. These results suggest that short-term delays during early treatment, particularly in the real-world setting, may not compromise the curative potential of frontline therapy in HL. To the best of our knowledge, this is the first study to evaluate both DTI and the first IcD as independent prognostic variables in a homogeneously treated cohort of HL patients receiving ABVD.

The favorable prognosis of HL, even in advanced stages, is well established and may explain this resilience to modest treatment delays [13]. Unlike aggressive lymphomas such as DLBCL or mantle cell lymphoma (MCL), where shorter DTI has been correlated with inferior outcomes due to high-risk clinical features, HL often follows a more indolent course. In MCL, for instance, Epperla et al. demonstrated that patients initiating treatment within 14 days of diagnosis had significantly worse PFS and OS, largely due to higher tumor burden and poor performance status at baseline [14]. Similarly, studies in DLBCL, including those by Maurer et al. and Yoshida et al., found that a short DTI independently predicted poorer PFS and OS, even after adjusting for known prognostic variables [7,8]. Interestingly, a recent multicenter U.S. study by Epperla et al. extended this investigation to marginal zone lymphoma (MZL), a more indolent lymphoma subtype [15]. The authors found that a shorter DTI in MZL patients was associated with the presence of B symptoms but did not adversely impact PFS or OS. These results suggest that in less aggressive lymphomas such as MZL and HL, DTI may reflect clinical presentation rather than serve as an independent prognostic factor.

The role of IcD, particularly delays after the first cycle of chemotherapy, is less well defined in HL. In our cohort, approximately 24% of patients experienced a delay exceeding 14 days between the first and second cycles, with neutropenia being the most common contributing factor. Importantly, these delays were not associated with adverse survival outcomes. This contrasts with studies of other malignancies, where chemotherapy delays and reduced dose intensity have been associated with worse outcomes [16,17]. Outcomes for older individuals (over 60 years of age) with HL tend to be less favorable compared with those of younger patients [18]. Although there are some biological differences between younger and older patients, the most important issue remains the toxicity of ABVD [19]. Infections are particularly crucial for older patients with HL, as they may be more vulnerable to complications. The risk of infection during neutropenia in our cohort was extremely low; thus, it may be better not to delay chemotherapy in case of neutropenia, especially in young patients. On the contrary, patients over 60 years of age should be given significant attention, especially for toxicities, as novel agents are increasingly integrated into their treatment regimen [20].

Our rationale for evaluating only the first IcD was guided by clinical considerations, as early cycles are most prone to treatment-limiting toxicities, and by precedent in emerging prognostic tools such as the Delay-7 score [21]. The Delay-7 model, introduced by Perrier et al., was derived from a large French national registry of patients with aggressive lymphomas receiving curative-intent immunochemotherapy. It incorporates seven binary variables, including age over 60, advanced stage, poor performance status, elevated LDH, hypoalbuminemia, extranodal disease, and a first IcD of more than three days. Notably, even a brief delay (>3 days) between the first two cycles carried independent prognostic value and was weighted equally to established risk factors in predicting survival outcomes. This discrepancy likely reflects fundamental biological differences between HL and aggressive NHL subtypes. Furthermore, in our cohort, no patients required dose reductions, and G-CSF use was not assessed. The routine use of G-CSF with ABVD remains controversial due to the potential risk of bleomycin-related pulmonary toxicity [22]. Only a small proportion of delays were temporally associated with the COVID-19 pandemic, indicating that most interruptions were attributable to clinical toxicity or logistical factors. The observation that over half of the patients received their second cycle on schedule supports the real-world feasibility of timely ABVD delivery.

In addition to treatment timing, we evaluated the prognostic role of baseline clinical and laboratory features. The prognosis of HL remains excellent across both early and advanced stages, particularly when risk-adapted strategies are applied. In early-stage disease, favorable prognostic factors include the involvement of a limited number of lymph node regions, absence of B symptoms, low ESR, and absence of bulky disease [23]. For advanced-stage HL, the International Prognostic Score (IPS) is widely used and incorporates seven variables: age ≥ 45 years, male gender, stage IV disease, hemoglobin < 10.5 g/dL, albumin < 4 g/dL, leukocytosis (≥15,000/mm^3^), and lymphocytopenia (<600/mm^3^ or <8% of the white blood cell count) [24]. In our cohort, as expected, PFS was associated with younger age, higher hemoglobin levels, and elevated ESR. OS was poorer in older patients and in those with lower hemoglobin, lymphocyte count, and albumin levels. Of particular interest, elevated beta-2 microglobulin emerged as an independent predictor of both PFS and OS in our study. Beta-2 microglobulin is a well-known prognostic factor in multiple myelomas and some hematological malignancies [25,26]. The role of beta-2 microglobulin levels in HL was first studied by Dimopoulos et al. They revealed that higher levels of beta-2 microglobulin were associated with advanced stage and poorer prognosis [27]. This marker, though not incorporated into the IPS, has previously been validated as a prognostic indicator in HL and may serve as a useful adjunct to conventional risk stratification tools [28]. Future studies may consider integrating beta-2 microglobulin or other serologic markers into modified prognostic algorithms tailored for the current treatment era.

This study uniquely focuses on the prognostic relevance of both DTI and the first IcD in patients with HL treated homogeneously with ABVD. To the best of our knowledge, this represents the first study focused on the evaluation of both variables in the HL population. The inclusion of a well-defined, real-world cohort with uniform treatment and long-term follow-up enhances the clinical relevance of the findings. Limiting the analysis to the first IcD provides a clearer assessment of early treatment dynamics, which are most susceptible to toxicity-related disruptions. However, our study has some limitations. The retrospective and single-center design may introduce selection bias and limit the generalizability of the results. The study was also conducted during a period that overlapped with the COVID-19 pandemic, which may have introduced non-medical delays in treatment unrelated to disease or toxicity. Finally, as the therapeutic landscape of HL continues to evolve with the integration of novel agents, findings from this study may not be fully applicable to current or future treatment paradigms.

## 5. Conclusions

In this study, neither DTI nor the first IcD was associated with PFS or OS in patients with HL treated with ABVD. These findings suggest that modest delays in initiating or continuing therapy may not adversely affect long-term outcomes in this curable malignancy. Among routinely assessed clinical parameters, age, hemoglobin level, ESR, and beta-2 microglobulin emerged as significant prognostic markers. Given the evolving treatment landscape, future prospective studies are warranted to validate these observations and to further elucidate the interaction between treatment timing and emerging prognostic factors in HL.

## Figures and Tables

**Table 1:** Baseline characteristics of patients (n = 137).

Characteristics		
Age at diagnosis *		34 (18–74)
Gender	Male, n (%)	85 (62%)
	Female, n (%)	52 (38%)
IcD, day *		0 (0–6)
DTI, day *		14 (7–27)
Hemoglobin, g/dL **		12.7 ± 2.1
Leucocytes, 10^9^/L *		8.6 (6.7–11.9)
Lymphocytes, ×10^9^/L *		1.6 (1.1–2)
Beta-2 microglobulin, ng/mL *		1700 (1379–2230)
ESR, mm/h *		34 (15–63)
Albumin, g/dL *		4.2 (3.6–4.4)
Bulky disease, n (%)		21 (15.3%)
B symptoms, n (%)		65 (47.4%)
Stage, n (%)	1	14 (10.2%)
	2	59 (43.1%)
	3	30 (21.9%)
	4	34 (24.8%)
Hasenclever score for patients with stage 3–4, n (%) (N = 64)	0	4 (6.3%)
	1	13 (20.3%)
	2	17 (26.6%)
	3	14 (21.9%)
	4	13 (20.3%)
	5	3 (4.6%)
Risk status for patients with stage 1–2, n (%) (N = 73)	Favorable	54 (74%)
	Unfavorable	19 (26%)

DTI: Diagnosis Treatment Interval; ESR: Erythrocyte sedimentation rate; IcD: Inter-Cycle Delay; * Median and interquartile range; ** Mean ± standard deviation.

**Table 2:** Multivariable analysis of progression-free survival.

	HR (95% CI)	*p*-Value
Age	0.951 (0.908–0.996)	**0.034**
Gender (Male vs. Female)	1.941 (0.672–5.603)	0.220
Hemoglobin, g/dL	0.969 (0.722–1.301)	0.835
Lymphocytes, ×10^9^/L	0.759 (0.345–1.668)	0.492
Beta-2 microglobulin, ng/mL	1.001 (1.000–1.002)	**0.018**
Albumin, g/dL	0.859 (0.289–2.551)	0.784
ESR, mm/h	0.992 (0.974–1.011)	0.43

CI: confidence interval, ESR: erythrocyte sedimentation rate, HR: hazard ratio. Boldface values signify statistically significant values.

**Table 3:** Multivariable analysis of overall survival.

	HR (95% CI)	*p*-Value
Age	1.061 (1.000–1.125)	**0.049**
Hemoglobin, g/dL	0.921 (0.567–1.496)	0.740
Leucocyte ×10^9^/L	0.855 (0.652–1.12)	0.256
Lymphocytes, ×10^9^/L	0.987 (0.289–3.371)	0.984
Beta-2 microglobulin, ng/mL	1.001 (1.000–1.001)	**0.037**
Albumin, g/dL	0.577 (0.191–1.742)	0.329
ESR, mm/h	1.01 (0.977–1.043)	0.562

CI: confidence interval, ESR: erythrocyte sedimentation rate, HR: hazard ratio. Boldface values signify statistically significant values.

## Data Availability

The data that support the findings of this study are available from the corresponding author upon reasonable request.
